# RGD-Dependent Epithelial Cell-Matrix Interactions in the Human Intestinal Crypt

**DOI:** 10.1155/2012/248759

**Published:** 2012-09-05

**Authors:** Yannick D. Benoit, Jean-François Groulx, David Gagné, Jean-François Beaulieu

**Affiliations:** ^1^Département d'Anatomie et Biologie Cellulaire, Faculté de Médecine et des Sciences de la Santé, Université de Sherbrooke, Sherbrooke, QC, Canada J1H 5N4; ^2^Pharmacology Department, Weill Cornell Medical College, New York, NY 10065, USA

## Abstract

Interactions between the extracellular matrix (ECM) and integrin receptors trigger structural and functional bonds between the cell microenvironment and the cytoskeleton. Such connections are essential for adhesion structure integrity and are key players in regulating transduction of specific intracellular signals, which in turn regulate the organization of the cell microenvironment and, consequently, cell function. The RGD peptide-dependent integrins represent a key subgroup of ECM receptors involved in the maintenance of epithelial homeostasis. Here we review recent findings on RGD-dependent ECM-integrin interactions and their roles in human intestinal epithelial crypt cells.

## 1. Introduction

Cell contacts with the extracellular matrix (ECM) provide both cohesive and functional properties in a variety of tissues, such as epithelia, nerves, muscle, and stroma, through specific interactions with cell membrane receptors [1, 2]. All ECMs are made up of collagen fibrils and/or networks, proteoglycans as well as specialized glycoproteins such as fibronectin and laminins that are archetypal of interstitial ECM and basement membrane (BM), respectively [3, 4]. Cells from multiple origins interact with ECM molecules using a variety of receptors, most of them being members of the integrin superfamily [2]. Integrins are noncovalent transmembrane *α*/*β* heterodimers. In mammals, over 24 distinct integrin heterodimers have been characterized to date, describing the association between 18*α* and 8*β* subunits [5–7]. The fact that integrin-mediated connections between the ECM and the cytoplasm regulate key cell functions such as adhesion, migration, proliferation, apoptosis, and differentiation is well recognized [8–11].

**Figure 1:**
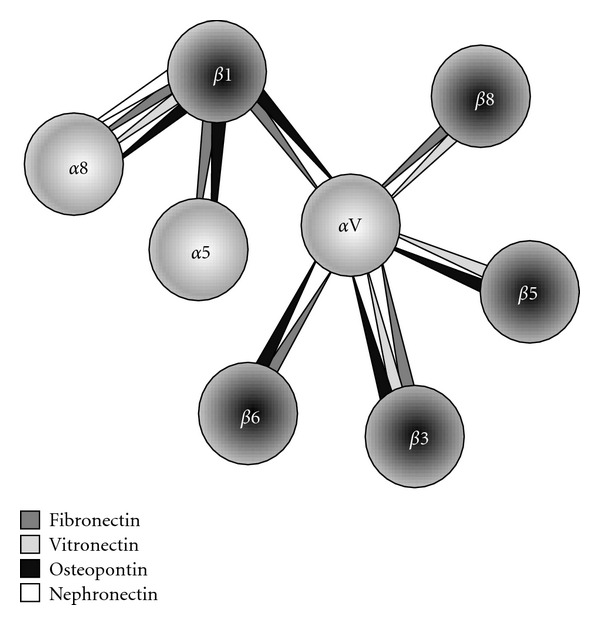
The RGD-dependent integrins. The RGD peptide (Arg-Gly-Asp) binding integrins represent a subclass of integrin receptors that specifically interact with the RGD motif found in several ECM elements. RGD integrins are formed by *α*8/*α*5 subunits coupled with the *β*1 subunit and the *α*V subunit coupled with *β*3/*β*5/*β*6/*β*8 subunits. RGD-dependent *α* and *β* heterodimers are connected to each other with respect to their specific RGD containing ligands. The major RGD ligands are fibronectin (dark gray), vitronectin (light gray), osteopontin (black), and nephronectin (white).

Epithelia express a wide variety of typical integrin receptors such as the *α*1*β*1, *α*2*β*1, *α*3*β*1, and *α*6*β*4 integrins that serve as collagen and/or laminin receptors [12–15]. Although less well documented in epithelia, the RGD-dependent integrins are another group of receptors that appears to be involved in epithelial cell homeostasis [15–17]. RGD-dependent integrins include *α*5*β*1-, *α*8*β*1-, and *α*V-containing integrins and are named as such because they specifically recognize the RGD motif, a sequence of three amino acids (Arg-Gly-Asp) found in many ECM molecules such as fibronectin and osteopontin [5, 12, 14, 15]. Collectively, these interactions are termed the “RGD-dependent adhesion system” (Figure 1). Interestingly, RGD-dependent cell interactions represent a key role in hierarchical assembly and maturation of adhesion structures including focal complexes (FXs), focal adhesions (FAs), and fibrillar adhesions (FBs) [1, 2, 18].

Therefore, RGD adhesion can by divided into three distinct components, the extracellular component (e.g., fibronectin), the membrane receptor (e.g., the *α*5*β*1 integrin), and the intracellular molecule (e.g., vinculin). Moreover, each component acts in concert with the others to organize and regulate RGD adhesion dynamics. In this paper, we will focus on the importance of the RGD-dependent adhesion system for human intestinal crypt cell homeostasis (Section 2). We chose to elaborate on recent findings from our laboratory related to each of the RGD adhesion components, the *α*8*β*1 integrin (receptor, Section 3), integrin-linked kinase (ILK) (intracellular molecule, Section 4), and type VI collagen (ECM, Section 5). 

## 2. Cell-Matrix Interactions in the Human ****Intestinal Crypt

**Figure 2:**
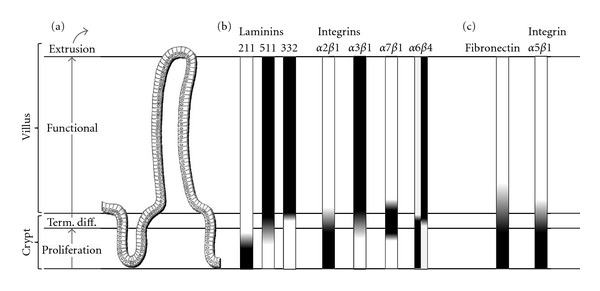
Distribution of laminins and laminin receptors of the integrin family as well as the RGD components fibronectin and the *α*5*β*1 integrin receptor along the crypt-villus axis in the human small intestine. (a) Organization of the crypt-villus epithelial renewing unit. Villi are lined by functional epithelial cells responsible for digestion and absorption of nutrients. Stem cells located at the bottom of the gland generate transit amplifying cells that expand in the middle of the gland until they reach the upper gland region where they stop proliferating and undertake their terminal differentiation program before reaching the base of the villus. (b) Patterns of distribution of laminins at the epithelial BM as well as laminin receptors of the integrin family revealed differential expression of these molecules along the crypt-villus axis according to the cell state as shown by dark areas. (c) The RGD components fibronectin and its specific integrin receptor *α*5*β*1 were found mostly confined to the crypt region (dark areas).

The small intestinal epithelium is a useful model to investigate the relationship between cell state and interaction with the ECM because of the well-defined architecture of its renewing unit, the crypt-villus axis. Indeed, proliferative cells, differentiating cells, and mature functional cells are topologically restricted to distinct compartments: the lower two-thirds of the crypt, upper third of the crypt, and villus, respectively. Gene expression in intestinal crypt cells must therefore be tightly regulated to efficiently control stemness, proliferation, migration, and differentiation in order to ensure the right equilibrium for the production of functional cells destined to renew the villus epithelium [19, 20]. There is strong evidence that cell-matrix interactions are involved in the regulation of these cell functions in the crypt [12, 21, 22]. For instance, differential spatial expression of laminins in the epithelial BM and their epithelial integrin receptors were observed along the crypt axis while in vitro studies have revealed functional relationships between laminin-binding integrins and specific intestinal cell functions such as proliferation, migration, and differentiation [23–30]. A schematic illustration of the human crypt-villus axis and the spatial expression of laminins, laminin receptors of the integrin family, and the two classic RGD components fibronectin and the *α*5*β*1 integrin (as depicted by dark areas) is shown in Figure 2. Moreover, another example is the transient expression of the tenascin and osteopontin receptor *α*9*β*1 integrin in the lower third of the crypt of the immature small intestine as well as in proliferative epithelial crypt cells [31] and its reexpression in colon adenocarcinoma cells [32]. 

The RGD archetype fibronectin is another ECM component that was found strongly expressed in the epithelial BM of the crypts in both human and small laboratory animals [33–36]. Synthesis and deposition of fibronectin by proliferating intestinal epithelial cells was confirmed in vitro [26, 34]. Furthermore, expression of the fibronectin receptors, *α*5*β*1 and *α*V-containing integrins, was found to be associated with intestinal cell proliferation [21, 29, 37]. Taken together, these observations suggest that fibronectin may significantly contribute to the RGD system regulating intestinal crypt cell functions. 

To investigate this hypothesis, we used a strategy combining expression studies in the intact human intestine and functional studies using HIEC cells, a human intestinal epithelial crypt cell model well-characterized for the expression of typical features of intestinal crypt cells [38–41]. As summarized in the next sections, this experimental approach has led to the identification and characterization of new components of the RGD-dependent adhesion system that emphasize the importance of this adhesion system in human intestinal crypt homeostasis.

## 3. Integrin *α*8*β*1 as a Crucial Mediator ****of Crypt Cell-Matrix Interaction

### 3.1. Integrin *α*8*β*1 Is a Novel Regulator of Epithelial Cell Adhesion

**Figure 3:**
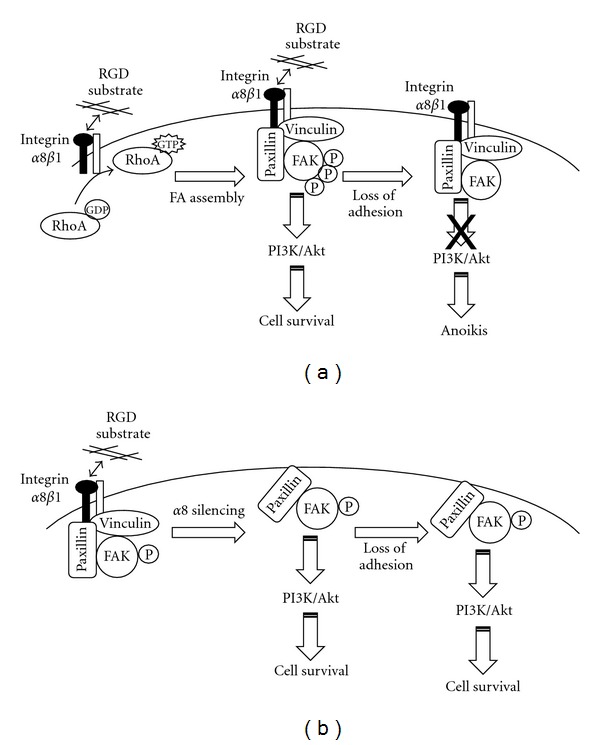
RGD-dependent adhesion influences anoikis sensitivity in undifferentiated epithelial cells. (a) Schematic representation of the proposed mechanism by which integrin *α*8*β*1 and RGD-dependent adhesion regulates anoikis sensitivity, through differential interactions between vinculin, paxillin, and FAK in intestinal epithelial crypt cells. As described in Section 3, *α*8*β*1 is essential to vinculin recruitment within maturing adhesion structures, while paxillin localization is not affected by *α*8 subunit silencing. (b) Following *α*8 subunit silencing, the absence of vinculin, combined with the presence of paxillin in the adhesion complexes, leads to an anchorage-independent activation of FAK and, consequently, anoikis resistance.

Initially characterized in the chicken nervous system [42, 43], integrin *α*8*β*1 represents an important RGD-dependent receptor [44]. Ligand binding to integrin *α*8*β*1 was shown to be important for RhoA GTPase activation and subsequent actin stress fiber assembly in vascular smooth muscle cells [45–47]. Integrin *α*8*β*1 was also recently found to play an important role in microfilament organization which was central to RGD-dependent intestinal epithelial crypt cell adhesion [48]. *α*8 subunit knockdown experiments, carried out in HIEC cells, showed that this integrin is important for proper vinculin recruitment to adhesion structures [48] (Figure 3). Intestinal epithelial crypt cells in which *α*8 was knocked down exhibited lower numbers of vinculin-positive FAs compared to controls, while paxillin localization was not affected [48]. It is well known that RhoA/ROCK signalling enhances actin stress fiber assembly and increases cell adhesion [49–51]. RhoA activity was shown to promote scaffolding protein recruitment, including vinculin, to the developing adhesion structures [51, 52]. Thus, the increased RhoA activity displayed by *α*8 knockdown cells leads to the absence or reduced levels of vinculin observed within these cells [48, 53]. 

Based on the scheme of adhesion structures hierarchical assembly, vinculin recruitment occurs at later stages of FX formation, while paxillin is recruited at early stages [54]. Thus, observations made in intestinal epithelial crypt cells suggest that integrin *α*8*β*1 is essential, at this particular stage of FX maturation into FA, via its role in RhoA activation [48] (Figure 3(a)). A similar function could also be predicted for the collagen-binding integrin *α*2*β*1, considering the expression of this receptor in undifferentiated intestinal epithelium cells and its participation in RhoA activation [44, 55, 56]. 

Interestingly, ectopic expression of the enterocytic differentiation associated factor GATA-4 in intestinal epithelial crypt cells caused a depletion of *α*8 subunit expression [39, 48]. In these same cells, reduced levels of *α*8*β*1 were associated with a decrease in cell growth, marked by Cyclin D1 inhibition and accumulation of cells in the G1 phase [48]. Similarly, decreased RhoA activity was observed in differentiated and nonproliferative HT29 cells compared to their undifferentiated and proliferative counterparts [55]. Together with the role of integrin *α*8*β*1 in RGD-dependent adhesion, these findings support the concept that cell-ECM interactions are crucial to maintaining a proliferative state in epithelial cells, which is anchorage and cell position dependent, reflecting its exclusive localization in the lower crypt of the intact intestine. 

### 3.2. Integrin *α*8*β*1 Regulates Crypt Cell Migration

Due to the role of integrin *α*8*β*1 in RGD-dependent cell adhesion and RhoA GTPase activity, this receptor was shown to exert a critical influence on intestinal epithelial crypt cell motility [48]. Alteration of RhoA activity was found to modulate migration in different systems [49, 57]. We recently reported that loss of RGD-*α*8*β*1 interactions in intestinal epithelial cells caused increased cell migration [48]. From a physiological perspective, proliferating intestinal epithelial cells must be restricted to the lower two-thirds of the crypt to avoid premature terminal differentiation and loss of proliferative capacity [38, 40]. Therefore, without necessarily affecting the expression of differentiation master regulator genes, RGD-dependent adhesion plays a major role in regulating cell migration, which in turn is crucial for wound healing, cell differentiation, and tissue integrity [24, 58, 59].

### 3.3. Integrin *α*8*β*1 RGD-Dependent Interactions Act as a Check Point in the Intestinal Crypt Epithelium

Cell survival is tightly regulated by RGD-dependent ECM-integrin interactions [11]. Indeed, integrin receptors, such as *α*5*β*1 and *α*V integrins, play a central role in controlling anoikis or apoptosis by loss of attachment [10, 11, 60]. Specifically, engagement of *β*1 integrins was found to be essential to intestinal epithelial cell survival through FAK signalling [60, 61]. 

As mentioned above, integrin *α*8*β*1 is involved in efficient vinculin recruitment to developing adhesion structures [48, 53]. The presence of vinculin in cell-ECM adhesion structures affects cell survival signal transduction. As previously described, HIEC cells share a number of features with intestinal epithelial stem cells, including a proliferative and undifferentiated state as well as the expression of several putative stem cell markers [39]. Interestingly, silencing of vinculin expression in F9 embryonic teratocarcinoma cells, another cell model closely related to stem cells [62], has shown increased resistance to anoikis, while ectopic reexpression of vinculin restored sensitivity to anchorage-dependent survival [63]. A similar phenomenon was observed in nonadherent *α*8 knockdown intestinal epithelial crypt cells [53]. In both studies, elevated levels of FAK phosphorylation on tyrosine 397 were noted in nonadherent cell cultures. The absence of vinculin combined with the presence of paxillin in primitive adhesion structures prior to loss of adhesion could explain such a phenomenon (Figure 3). At the molecular level, it has been shown that paxillin exhibits partially overlapping binding sites for FAK and vinculin [64]. Thus, the vinculin tail domain appears to compete with FAK for paxillin binding. In the presence of vinculin, FAK activation would depend on ECM-integrin binding. However, absence of vinculin leads to a conformational change in adhesion structures which results in constitutive activation of FAK when bound to paxillin [63] where FAK activity no longer relies on ECM-integrin interactions [53, 63]. Additionally, nonadherent *α*8*β*1-depleted intestinal epithelial cells showed increased activity of the PI3 K/Akt signalling pathway compared to nonadherent controls [53]. A summary of integrin *α*8*β*1 contribution to anoikis regulation in epithelial intestinal crypt cells is presented in Figure 3. 

Considering the proliferative and highly adaptive capacities of crypt cells, such as stem and transit amplifying cells, RGD-dependent *α*8*β*1 interactions with ECM are suggested to act as a security switch that keeps the detachment of undifferentiated epithelial cells in check. It is worth noting that none of the five human colorectal cancer cell lines tested were found to express the integrin *α*8 subunit and that ectopic expression of this RGD-dependent receptor restored sensitivity of malignant cells to anoikis [53]. The mechanism by which colon cancer cells repress *α*8 expression to bypass this checkpoint remains unknown. However, in normal cells, this security step mediated by *α*8*β*1 occupancy is potentially important to support homeostasis in the human intestinal crypt. New evidence from the literature has shown that colon cancer may originate from defective crypt stem cells [65]. Therefore, in light of the expression of *α*8*β*1 in the region associated with intestinal stem cells, combined with its role in sensitizing epithelial cells to anoikis [53], it could be speculated that *α*8 integrin silencing represents a key step in cancer initiation, in order to escape apoptosis upon modification of the malignant stem cell niche or location. In this context, *α*8*β*1 could promote defective progenitor cell elimination, and consequently prevent the onset of ecto-cryptal proliferative structures. Such specific involvement of RGD-dependent integrins is not without precedent since altered expression of other heterodimers has been reported in colon cancer. For instance, integrin *α*V*β*3 expression has been found to be specifically decreased in anoikis-resistant Caco-2 cells [66]. 

## 4. Integrin-Linked Kinase (ILK) as an ****Integrator of Cell-Fibronectin Interaction

The intestinal epithelial cell mediates RGD interactions through expression of specific integrin receptors, as observed with *α*8*β*1, as well as through the production and deposition of RGD ligands, such as fibronectin. The efficient deposition of fibronectin into the BM relies upon its recognition by RGD-dependent integrins, which mediate its unfolding to expose specific fibronectin structural domains, which in turn mediates the formation of insoluble fibronectin fibrils [67]. Fibronectin deposition is characterized by the formation of specialized cell-matrix contact structures containing integrins, cytosolic proteins, and actin referred to as fibrillar adhesion (FB) points [67]. 

The integrin-linked kinase (ILK) is a constituent of integrin containing adhesion sites where it mediates multiple cellular processes. ILK is a pseudokinase and scaffolding protein ubiquitously expressed in mammalian cells forming a trimeric complex with PINCH and parvins named the IPP complex [68–70]. ILK interacts with the cytoplasmic domain of integrin *β*1 and *β*3 subunits to create a physical link between integrins and the actin cytoskeleton [68, 71]. Interestingly, it has been suggested that ILK regulates fibronectin expression/deposition [72–74] and other studies have placed IPP complex members within FA points [75, 76]. In vivo, fibronectin expression is restricted to the BM underlying epithelial crypt cells and HIEC cells produce copious amount of fibronectin and generate numerous well-defined adhesion structures. The expression and roles of ILK were therefore investigated in human intestinal crypt cells.

**Figure 4:**
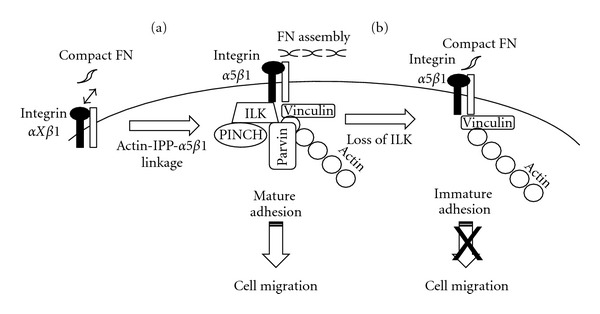
Regulation of the dynamic assembly of fibronectin by ILK. Schematic representation of the proposed mechanism by which ILK regulates FN assembly in HIEC. (a) Following cellular adhesion, the ILK/IPP complex is recruited to focal adhesions providing the link between the actin cytoskeleton and integrin *α*5*β*1. IPP recruitment mediates the formation of mature focal adhesions that allow fibronectin deposition into the BM of HIEC. (b) Depletion of ILK in HIEC results in the disruption of the IPP complex which prevents actin cytoskeleton-*α*5*β*1 linkage and focal adhesions cannot mature. Consequently, loss of the IPP complex reduces FN deposition and decreases migration of HIEC.

We first focused on the localization of ILK-related components in the small intestine. As previously observed for fibronectin and integrin *α*5*β*1 in the intact intestine [21, 29, 33], ILK, PINCH-1 *α*-parvin, and *β*-parvin were found to be predominantly expressed by the proliferative epithelial cells of the crypts [58]. In HIEC cells, ILK, PINCH-1, *α*-parvin, and *β*-parvin were all closely associated with FA points (Figure 4(a)). A siRNA strategy was used to knock down ILK expression in HIEC cells in order to further investigate the role of ILK in intestinal crypt cells [58]. Interestingly, ILK knockdown in HIEC was accompanied by severe disruption of the IPP complex including the loss of PINCH-1 and parvins as well as major alterations in fibronectin synthesis and functional matrix deposition (Figure 4(b)). Overexpression of ILK was previously shown to increase fibronectin deposition in rat intestinal cells [76] while ILK knockdown decreases fibronectin expression in mice and human colon cancer cells. Indeed, the fibronectin gene promoter contains response elements that have been shown to be potentially regulated by ILK-mediated signalling [68, 77]. However, in HIEC cells, although a reduction of fibronectin was observed at the transcript level, ILK knockdown had no net effect on fibronectin protein amounts found in the culture medium suggesting that it was mainly the ability to process and deposit soluble fibronectin that was altered by the loss of the IPP complex [58]. The exact mechanism by which ILK knockdown impairs fibronectin deposition remains to be elucidated. Expression levels of the fibronectin integrin receptors were not altered in HIEC ILK knockdown cells suggesting that the required receptors for fibrillogenesis [67] remain available for binding. However, because of the important scaffolding role of ILK and IPP complexes, the decrease of fibronectin deposition in these ILK-deficient cells may reflect a reduction in the cytoskeletal tension necessary for fibrillogenesis [78, 79]. Alternatively, alteration in signalization pathways may also be involved. Indeed, key signalling molecules such as Src, PI3 K, and ERK have been shown to modulate fibronectin deposition in various cell models [80–82] and ILK and the IPP complexes can regulate these signalling molecules [83–85].

In addition to an alteration in fibronectin deposition, ILK knockdown severely affected basic intestinal crypt cell functions such as cell spreading, migration restitution abilities as well as cell proliferation [58]. Alterations in these functions in ILK-knockdown HIEC cells were not surprising since these functions can be stimulated by fibronectin in intestinal epithelial cells [86–89]. Interestingly, exogenously deposited fibronectin was found to fully rescue the ILK-knockdown HIEC phenotype with regard to cell proliferation, spreading and migration [58].

Taken together, as summarized in Figure 4, these data reveal that ILK and, by extension, the IPP complexes, perform crucial roles in the control of human intestinal crypt cell homeostasis, especially as key mediator of fibronectin deposition in the BM, which in turn regulates cell proliferation, migration, and restitution. 

## 5. BM Collagen VI as a Regulator of Crypt-Cell-Fibronectin Interaction

Type VI collagen is a ubiquitously expressed ECM component [90]. In interstitial ECM, collagen VI acts as an anchoring meshwork bridging collagen fibers to the surrounding matrix [91, 92]. Collagen VI has also been shown to directly interact with the BM-specific type IV collagen [93] supporting a key role for this collagen in connecting BM to ECM [94, 95]. However, we recently identified collagen VI as a *bona fide* component of the basal lamina in the intestinal BM and found that it is synthesized in significant amounts by crypt epithelial cells [59]. 

**Figure 5:**
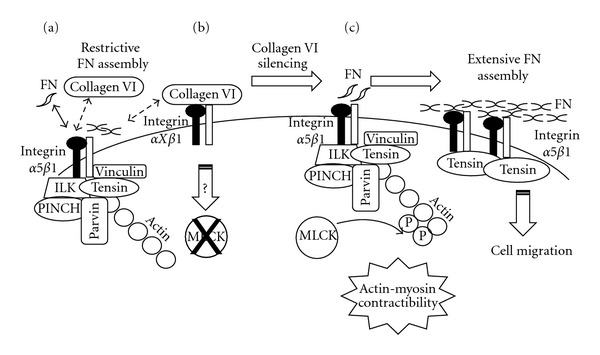
Regulation of dynamic assembly of fibronectin by type VI collagen. Schematic representation of the proposed mechanism by which collagen VI regulates FN fibrillogenesis. In HIEC, collagen VI is deposited into the ECM and interferes with fibronectin assembly by three distinct mechanisms. (a) First, in HIEC collagen VI competes with fibronectin for *β*1 integrin binding in focal adhesions. (b) Second, collagen VI limits cellular accessibility of fibronectin through a direct interaction with FN preventing fibronectin association with other fibronectin molecules, a step required for the extensive formation of the fibronectin matrix. (c) The third mechanism involves the regulation of MLCK by collagen VI. When collagen VI is depleted from the ECM, the MLCK/MLC pathway is activated by an unknown mechanism and mediates fibrillar actin contractility that allows the recruitment of tensin to FBs generating extensive fibrillogenesis. The increase in fibronectin deposition followed the depletion of collagen VI is accompanied by an increase in migration.

To investigate the function of type VI collagen in the intestinal epithelial crypt cell, we used a similar knockdown strategy with HIEC cells as described in the previous sections for integrin the *α*8 subunit and ILK. Surprisingly, abolition of collagen VI expression resulted in a striking increase in cell size and spreading accompanied by a significant increase in the number of stress fibers and tensin recruitment at the FB points [59]. The observations that removal of collagen VI emphasized features normally associated with fibronectin suggested that collagen VI regulates fibronectin assembly in epithelial cells. Interactions between collagen VI and fibronectin have been previously reported [93, 96]. Further investigation in collagen-VI-depleted HIEC cells revealed that fibronectin was increased at both protein and transcript levels and was subjected to extracellular rearrangement into long, parallel fibrils. Importantly, exogenous collagen VI, but not collagen I or IV, was able to fully rescue the knockdown phenotype indicating that the effect is specific for type VI collagen [59]. Considering that exposure of fibronectin-binding sites is critical for both cell binding and fibrillogenesis [67, 97], one may hypothesize that, under normal conditions, collagen VI acts by limiting cellular accessibility to fibronectin through competition for integrin receptors (Figure 5(a)) or by a direct interaction with fibronectin in the ECM (Figure 5(b)). Consistent with this possibility, collagen VI has been reported to be recognized by the RGD-binding *α*5*β*1 and *α*V integrins [98–100]. Furthermore, HIEC binding to collagen VI is integrin *β*1 dependent and it was the FB complexes, specifically enriched in tensin and *α*5*β*1 integrin [6, 54, 67], that were enhanced in collagen-VI-depleted HIEC [59]. 

To further investigate the mechanism underlying the generation of FB complexes in collagen-VI-depleted intestinal crypt cells, the regulation of actomyosin forces was analyzed. Actin contractility depends on the phosphorylation of the myosin light chain (MLC), which is mainly mediated by the kinases MLC (MLCK) and Rho (ROCK) acting on MLC and myosin phosphatase, respectively [101–104]. Interestingly, MLCK-dependent activation of MLC phosphorylation was observed in poor collagen VI/rich fibronectin ECM environments consistent with the observed generation of higher numbers of tensin-enriched FB complexes and extensive fibronectin fibrillar deposition [59] (Figure 5(c)).

As summarized in Figure 5, these data identified collagen VI as a major regulator of fibronectin synthesis and fibrillogenesis and suggest that collagen VI influences intestinal epithelial crypt cell behaviour by restraining cell-fibronectin interactions and their downstream events. 

## 6. Conclusions

Described as a predominant epithelial BM component in the intestinal crypt more than 3 decades ago [33–36], fibronectin has been confirmed to play an important role in the RGD system regulating crypt epithelial cell functions. The recent findings summarized herein further emphasize the crucial importance of this RGD-adhesion system and its regulatory mechanisms. Indeed, intestinal epithelial cells can regulate RGD interactions through expression of specific integrin receptors, as exemplified by *α*8*β*1, which exerts major regulatory influences on key cell functions such as cell proliferation, migration, and survival [48, 53]. Regulation of RGD interactions can also be accomplished by regulating production and deposition of their ligands, such as fibronectin, as illustrated by the finding that ILK/IPP complexes are key mediators of fibronectin deposition into the BM, which in turn regulates cell proliferation, migration, and restitution [58]. Finally, regulation of RGD-dependent cell interactions can also be achieved by interaction with other ECM molecules as shown with type VI collagen, a basement membrane component that regulates epithelial cell-fibronectin interactions. Taken together, these studies define new molecular elements and shed new light on the relative complexity of specific cell-matrix interactions in a well-defined environment such as the intestinal crypt and the critical impact these interactions have on cell function.
